# Construction and evaluation of immune-related diagnostic model in patients with heart failure caused by idiopathic dilated cardiomyopathy

**DOI:** 10.1186/s12872-023-03666-1

**Published:** 2024-02-06

**Authors:** Sichi Xu, Zhaogui Wu, Haihua Chen

**Affiliations:** 1 Department of Cardiology, The Central Hospital of Wuhan, Tong Ji Medical College, Huazhong University of Science and Technology, Wuhan, 430014 China; 2 Key Laboratory for Molecular Diagnosis of Hubei Province, The Central Hospital of Wuhan, Tong Ji Medica College, Huazhong University of Science and Technology, Wuhan, 430014 China; 3 Department of Cardiology, Xiangyang No.1 People’s Hospital, Hubei University of Medicine, Xiangyang, Hubei China; 4 Emergency Center, Zhongnan Hospital of Wuhan University, Wuhan, 430071 Hubei China; 5 Hubei Clinical Research Center for Emergency and Resuscitation, Zhongnan Hospital of Wuhan University, Wuhan, 430071 Hubei China

**Keywords:** Idiopathic dilated cardiomyopathy, Immune gene, Diagnosis, Heart failure

## Abstract

**Objective:**

The purpose of the study was to construct the potential diagnostic model of immune-related genes during the development of heart failure caused by idiopathic dilated cardiomyopathy.

**Method:**

GSE5406 and GSE57338 were downloaded from the GEO website (https://www.ncbi.nlm.nih.gov/geo/). CIBERSORT was used for the evaluation of immune infiltration in idiopathic dilated cardiomyopathy (DCM) of GSE5406. Differently expressed genes were calculated by the limma R package and visualized by the volcano plot. The immune-related genes were downloaded from Immport, TISIDB, and InnateDB. Then the immune-related differential genes (IRDGs) were acquired from the intersection. Protein–protein interaction network (PPI) and Cytoscape were used to visualize the hub genes. Three machine learning methods such as random forest, logical regression, and elastic network regression model were adopted to construct the prediction model. The diagnostic value was also validated in GSE57338.

**Results:**

Our study demonstrated the obvious different ratio of T cell CD4 memory activated, T cell regulatory Tregs, and neutrophils between DCM and control donors. As many as 2139 differential genes and 274 immune-related different genes were identified. These genes were mainly enriched in lipid and atherosclerosis, human cytomegalovirus infection, and cytokine-cytokine receptor interaction. At the same time, as many as fifteen hub genes were identified as the IRDGs (IFITM3, IFITM2, IFITM1, IFIT3, IFIT1, HLA-A, HLA-B, HLA-C, ADAR, STAT1, SAMHD1, RSAD2, MX1, ISG20, IRF2). Moreover, we also discovered that the elastic network and logistic regression models had a higher diagnostic value than that of random forest models based on these hub genes.

**Conclusion:**

Our study demonstrated the pivotal role of immune function during the development of heart failure caused by DCM. This study may offer new opportunities for the detection and intervention of immune-related DCM.

## Introduction

Cardiomyopathy belong to a heterogeneous condition that results in dilated, hypertrophic, or restrictive cardiomyopathy. Dilated cardiomyopathy (DCM) belongs to one of the most common and serious diseases which pose a huge threat to people’s life [1]. According to a previous statement, non-ischemic dilated cardiomyopathy (DCM) occupied almost 1 out of 2500 individuals in the general population and directly results in considerable morbidity and mortality [2]. Even worse, DCM predispose patients to end-stage refractory heart failure (HF) and even malignant ventricular arrhythmia (VA). The annual incidence of sudden cardiac death (SCD) in DCM has reached 2–4% which accounts for up to half of all the deaths [3, 4]. Currently, cardiomyopathies still account for a major proportion of heart transplantation worldwide. Although previous studies have achieved some advances in the field of diagnosis and treatment of HF, early diagnosis, prevention, and timely intervention of DCM still deserve further exploration.

The role of immune-mediated inflammatory response in the development and progression of DCM has been debated for many years. Direct evidence of cell-mediated immunity in DCM is validated by the frequent occurrence of lymphocyte and macrophage infiltration on EMB (Endomyocardial biopsy). The lymphocytic component has been validated by immunohistochemistry as being predominantly T-cell (CD3 +), while single-cell sequencing from explanted DCM hearts revealed that the proportion of exhausted/cytotoxic CD8 + and pro-inflammatory CD4 + T-cell subtypes [5] A large amount of evidence suggests that inflammation and immune dysregulation play a causative role in DCM as either a primary triggering factor or as a pivotal disease accelerator [6].

One of the most typical examples is myocarditis mediated DCM. Myocarditis can owe to a variety of causes and a heterogeneous clinical presentation characterized by immune cell infiltration in the myocardium. About one-third of patients will aggravate into DCM and the pathogenesis is complex and diverse, involving multiple immune disorder [7]. Although the disease is usually characterized with infiltration of the myocardium with either eosinophils or leukocytes, conventional treatment with immunosuppressants is not recommended due to limited effect in lymphocytic myocarditis depart from giant cell myocarditis. The moderate response from the host immunity may inhibit persistent virus replication in the heart so as to restraint the aggravation of the chronic myocarditis and DCM [8]. Deciphering the dynamic evolution and complexity of the immune response is pivotal to disease stratification and precise therapeutic strategy. The pathogenic process of myocarditis to DCM can be primarily divided into the three phases: the early rapid response of viral entry into the cell and the activation of the innate immune response which can lasted for 1–7 days, Then the subacute response accompanied with the activation of the adaptive immune response can maintain for almost 1–4 weeks. The final delayed and chronic phase that can last from months to years, in which the second or ineffective viral clearance together with chronic cardiac inflammation can finally result in the incidence of DCM [9].

Under normal circumstances, the early stage of immunity involves the innate immune system, when tissue damage and distress signals initiate rapid but nonspecific events. Simultaneously, the innate immune response triggers adaptive immunity, a specifically targeted inflammatory event involving T and B lymphocyte-mediated effects [10]. The endogenous triggers of tissue damaged in DCM usually include genetic mutation, alcohol abuse and viral and bacterial infections, while the exogenous triggers about the cardiac antigens including myosin, and troponin.

The nonspecific innate immune response were triggered through recognition by specific pattern recognition receptors, such as Toll-like receptors (TLRs), pathogen-associated molecular patterns (PAMPs), and damage-associated molecular patterns (DAMPs) [11]. Various immune cells were infiltrated in the heart ranging from granulocytes, macrophages and dendritic cells. These cells can release pro-inflammatory cytokines aiming at eliminating the noxious response caused by infection, pathogen, autoantigen and tissue necrosis [12]. Both the innate and adaptive immune response has aggravated the deterioration of DCM. The innate system activates the adaptive response by antigen presentation on major histocompatibility class II (MHC II) molecules. Cytokine release by the innate and adaptive systems can not only result in direct tissue damage, but also further trigger the innate/adaptive responses. A previous study has demonstrated that various inflammatory cells is closely related to the development of DCM, such as macrophage and T cells [13]. CD4 + T-helper (Th) cells play critical role in the interaction between innate and adaptive immune responses. They respond to antigen presented by MHC II molecules and can be mainly divided in to Th1, Th2, Th17 and Th22 functional subgroups. The over-activated innate and adaptive immune cells can be detectable in peripheral blood, lymphoid and cardiac tissue. The corresponding functional can be determined by the expression of different cluster of differentiation (CD) markers, including CD25, CD69 and CD45RO [14].

Moreover, the immune-related genes also take part in DCM-related HF. Elevated levels of cellular adhesion molecules, pro-inflammatory cytokines and macrophage-related proteins can be detected in patients with DCM and frequently indicating a poor prognosis. Multi animal studies has proposed that the direct myocardial effects of various pro-inflammatory cytokines (IL-1, IL-2, IL-6, tumor necrosis factor-α, TNF-α) has resulted in obvious impairment of contractility, LV dilatation and induction of endothelial dysfunction [15]. Significantly elevated numbers of circulating Th1, Th17 and Th22 cells have been detected in patients with DCM [16]. B cells are typically associated with the development of DCM via the secretion of antibodies against cardiac proteins. Moreover, they also play a critical role in antigen presentation and cytokine production and have been shown to exacerbate myocardial inflammation by suppressing the anti-inflammatory M2 macrophage [17]. EMB samples in DCM validate the presence of whole (CD68 +) and M2 (CD163 +) macrophages and higher macrophage infiltration imply the poorer prognosis [18]. In chronic DCM, neutrophil count and neutrophil to lymphocyte ratio (NLR) correlates with New York Heart Association (NYHA) functional class, BNP (brain natriuretic peptide) and left ventricular ejection fraction (LVEF). There are few human studies majoring in the role of mast cells, natural killer (NK) cells or eosinophils in DCM. Some authors have described a decreased activity of the NK cell subset, although this is relatively historical [19]. Eosinophils have been implicated in mouse model of acute myocarditis that progresses into DCM, which is dependent on the production of IL-4 [20]. Therefore, early recognition of immune-related genes is essential for the prevention of HF.

In recent years, machine learning has gained prominence for uncovering novel patterns from complex datasets compared to linear methods, which outperforms them in the long run. With the rapid accomplishment of high-throughput sequencing and bioinformatics analysis, an increasing number of biomarkers has been detected in the diagnosis and prevention of HF, such as middle regional adrenomedullin (MR-proADM), highly sensitive cardiac troponin I/T( cTnI/TnT), soluble ST2 (sST2), growth differentiation factor (GDF)-15, Galectin-3, H-FABP(Heart type-Fatty Acid Binding Proteins), and Cystatin C (Cys-C). However, these indicators also have certain limitations and are easily affected by renal function and body weight [21] (Table 1). In addition, different data sets accompanied by different analysis methods may generate different analyses even for the same datasets. Vijayakrishna Kolur concluded that HF is relevant to the adaptive immune system and neutrophil degranulation by analyzing GSE141910 and obtained 10 characteristic genes [22]. Comprehensive and in-depth analysis of immune-related genes (IRGs) is still relatively rare. In this study, we intend to develop prediction models based on differential expression in IRGs to predict the development of HF caused by DCM.

**Table 1:** The limitations of the common biomarkers in the HF

Biomarker	Influence factor
Pro-BNP	Renal function; sex; age; BMI
BNP	Recombinant human BNP; enkephalinase inhibitors
Pro-ANP	Renal function; sex; age; BMI
cTnI/TnT	Cardiogenic factors (coronary heart disease, myocarditis, aortic dissection, pulmonary heart disease, stress cardiomyopathy, cardiac amyloidosis, rapid ventricular arrhythmias, cardiac surgery, defibrillation, and heart trauma); Non cardiac factors include pulmonary embolism, renal insufficiency, stroke, infectious diseases (such as novel coronavirus pneumonia and sepsis), cardiac toxic injury of drugs, rhabdomyolysis, high-intensity exercise and burns
ST2	Pulmonary diseases (such as pneumonia, asthma, acute respiratory distress syndrome), liver diseases (such as liver cirrhosis, liver cancer), acute coronary syndrome, sepsis, trauma, stroke, tumors, autoimmune diseases
Pro-ADM	Vascular endothelial cell function; only predict short term prognosis of patients with heart failure
GDF-15	Inflammatory disease; diabetes; atherosclerotic cardiovascular disease
Gal3	Not suitable for acute heart failure
H-FABP	Only suitable for acute heart failure
Cys C	Inflammatory response; oxidative stress; renal function

## Methods

### Data collection

**Fig. 1:**
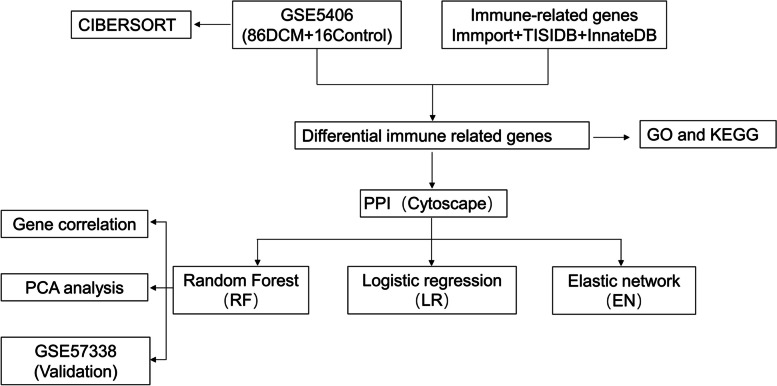
The primary network of this article

The two transcriptome profiling datasets, GSE5406 and GSE57338 were downloaded from the Gene Expression Omnibus database (GEO, http://www.ncbi.nlm.nih.gov/geo/). The dataset of GSE5406 included 16 control and 108 DCM tissue specimen data, while GSE57338 contains 82 DCM and 136 control. GSE5406 was used as the training dataset while GSE57338 was conducted as a validation dataset. Data of IRGs was downloaded from the ImmPort database (https://www.immport.org/shared/) [23], TISIDB (http://cis.hku.hk/TISIDB/) [24] and InnateDB (https://www.innatedb.ca/) database [25]. The workflow of the present study was shown in Fig. 1.

### Distribution of the immune cells in cardiac tissues

The relative proportion of immune cell types in samples from the GSE5406 dataset was calculated by using the CIBERSORT algorithm. Then, we compared and visualized the distribution of the 22 immune cells between DCM and control groups. Moreover, the relationship among 22 immune cell subtypes was presented by using the “corrplot” package.

### Differential expression and enrichment analysis

The differentially expressed genes (DEGs) between DCM and normal control were obtained by using the “Limma” package in R under the criteria of *P* < 0.05. Immune-related different genes (IRDGs) were acquired by the intersection between DEGs and IRGs by Venn Diagram. Gene Ontology (GO) analysis and Kyoto Encyclopedia of Genes and Genomes (KEGG) analysis of IRGs were calculated by using cluster profiler package R (4.0.2). Cluster Profiler refer to a package of R software that was intended to compare and visualize functional profiles among gene clusters. The top ten most prominent pathways were manifested and *P* value < 0.05 was considered to be significant difference.

### Construction of protein–protein interaction (PPI) network and hub gene identification

The PPI network was displayed by the Search Tool for the Retrieval of Interacting Genes (STRING) database. Cytoscape software (3.8.2) was used to visualize the PPI network. The hub genes were identified by the MCODE method. The relationship between hub genes was further visualized by Corrplot package R (0.90).

### Construction of diagnostic prediction models

The expressions of IRDGs were regarded as independent variables to construct a prediction model for the diagnosis of HF. Then, three different supervised machine learning algorithms were utilized to explore the diagnostic prediction model. The random forest model (RF), logical regression (LR), and elastic network model (EN) were respectively constructed. Then, the validation set GSE57338 was utilized to verify the prediction model, and the receiver operating characteristic curve (ROC) curve was adopted to evaluate the diagnostic ability.

### Identification of potential drugs

To further explore the candidate drugs for the treatment of DCM based on these hub genes. Drug Signatures database (DSigDB, http://biotechlab.fudan.edu.cn/database/drugsig/) [26] was adopted to investigate the potential drug via the friendly web-based enrichment analysis platform Enrichr website [27]. DSigDB acts as an online gene set linking drugs and their target genes were composed of 22527 gene sets, 17389 unique compounds, and 19531 genes [28]. *P* < 0.05 were considered statistically significant.

## Results

### Immune infiltration analysis

**Fig. 2:**
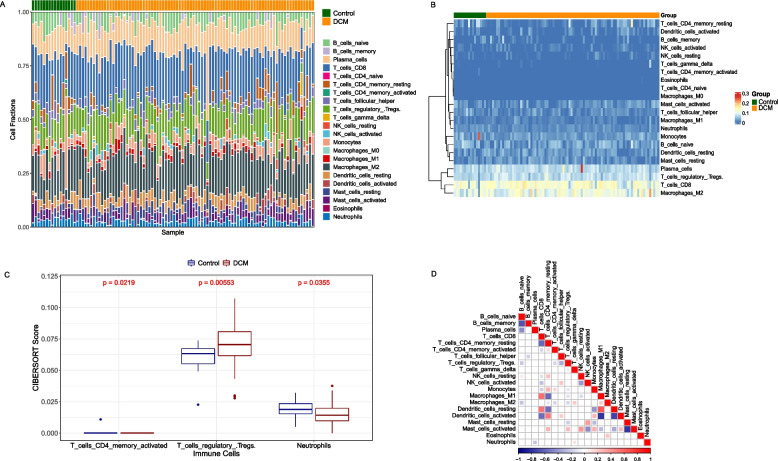
Analysis of the immunological landscape in GSE5406. **A** Immune cell proportions in samples from normal controls and DCM patients. **B** The visualization map of the total immune cell infiltration via heart map. **C** Comparison of the different immune cells in DCM patients and healthy samples. **D** The association between different types of immune cell infiltration

To provide a better understanding of the relationship between inflammation and DCM, the relative proportion of immune cell types in each sample was calculated in cardiac tissue. The specific proportion and distribution of the immune cells were visualized using the heat map and the histogram in Fig. 2A, B. Our study illustrated that the ratio of T cell CD4 memory activated, T cell regulatory Tregs, and neutrophils were significantly different from control donors (Fig. 2C). We also analyzed the correlation among different immune cells. It can be seen in Fig. 2D that macrophage-M1 were positively associated with T-cells-CD8 while T cells-CD4-memory-resting were negatively associated with macrophage-M1. Dendritic-cells-activated were negatively associated with macrophage-M1 and dendritic-cells-resting. The result demonstrated that various immune cells may take part in the progression of DCM.

### Identification of immune-related differential genes (IRDGs)

**Fig. 3:**
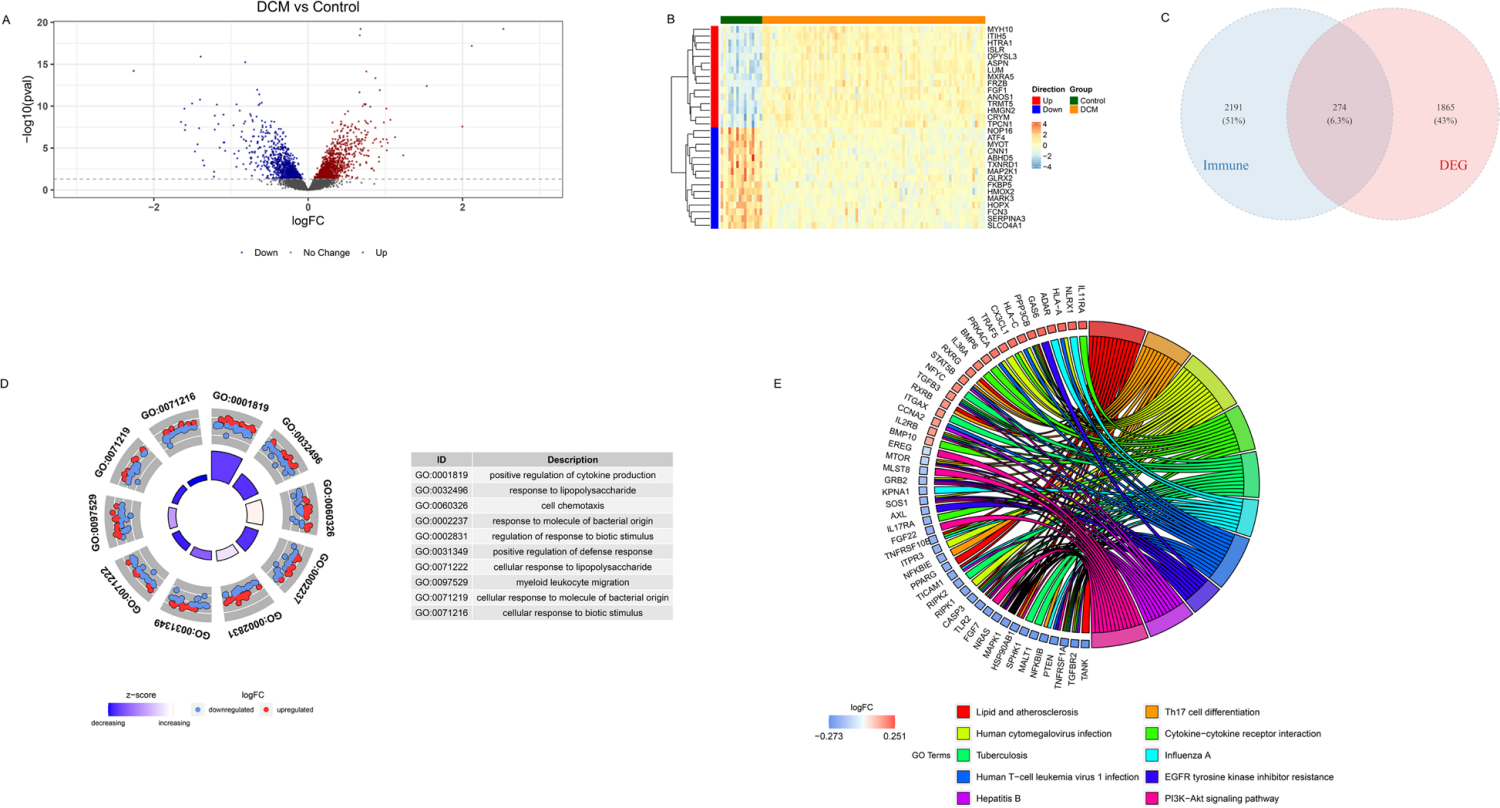
Identification of IRDG and enrichment analysis. **A** The volcano plot of the differential genes in DCM. **B** The heat map of the top 30 differential expressed genes between DCM and control samples. **C** The intersection between differential genes and IRGs. **D** GO enrichment analysis and the presentation of the top 10 pathways. **E** KEGG enrichment analysis of the top 10 pathways

Firstly, we identified the differential expressed genes between DCM and the control group. As many as 2139 differential genes between the two groups were identified by using the “Limma” package in R, of which 1169 genes were up-regulated, and 970 genes were down-regulated (Fig. 3A). The results of the top 15 up-regulated genes and 15 down-regulated genes with the most obvious difference were visualized in the form of heat map (Fig. 3B). Then we downloaded the immune-related genes from the ImmPort database, TISIDB, and InnateDB. The IRDGs are derived from the intersection of differential genes and genes related to immunity. As many as 274 IRDGs were obtained in the end (Fig. 3C).

### Functional analysis of IRDGs, construction of PPI network and Hub gene expression analysis

**Fig. 4:**
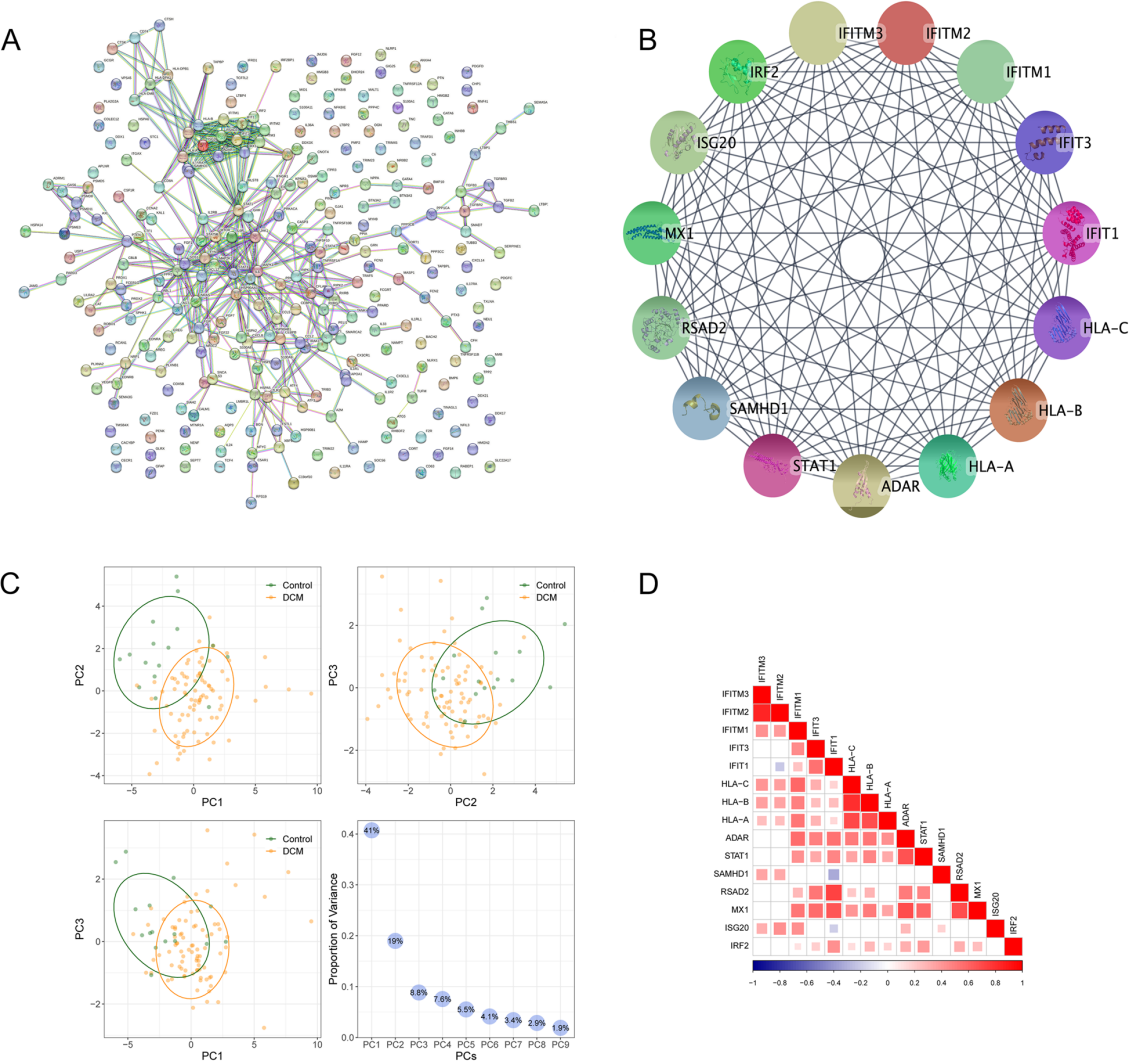
The identification of hub genes. **A** The PPI network of the IRDGs. **B** The top 15 hub genes. **C** The presentation of PCA based on these hub genes. **D** The correlation analysis based on hub gene expression

To further investigate the pathophysiology functions of these IRDGs in DCM, GO analysis according to the DAVID online database was adopted to interpret the biological process (BP), cell component (CC), and molecular function (MF). The results demonstrated that most genes participated in the positive regulation of cytokine production, response to lipopolysaccharide, and response to molecule of bacterial origin in BP. CC was most enriched in the secretory granule lumen, cytoplasmic vesicle lumen, and vesicle lumen. The MF was most enriched in receptor-ligand activity, signaling receptor activator activity, and cytokine receptor binding. The top ten most significantly enriched pathways were visualized by ring diagram (Fig. 3D). In addition, the KEGG pathway enrichment demonstrated that the three most obvious pathways were enriched in lipid and atherosclerosis, human cytomegalovirus infection, and cytokine-cytokine receptor interaction (Fig. 3E). The hub genes were visualized using a PPI network after processing by Cystoscope and MCODE (Fig. 4A). As many as fifteen hub genes were identified as the IRDGs (IFITM3, IFITM2, IFITM1, IFIT3, IFIT1, HLA-A, HLA-B, HLA-C, ADAR, STAT1, SAMHD1, RSAD2, MX1, ISG20, IRF2) (Fig. 4B). PCA (principal component analysis) was performed as a dimension reduction strategy. The results demonstrated that the DCM and the control groups could be well distinguished, which means that these differential genes might be acted as independent parameters for the diagnosis of DCM (Fig. 4C). We also depicted the interaction network of these hub genes (Fig. 4D). Such as SAMHD1 was negatively associated with IFIT1, while RSAD2 and MX1 were positively associated with IFIT1.

### Establishment of the diagnostic model based on IRDGs

**Fig. 5:**
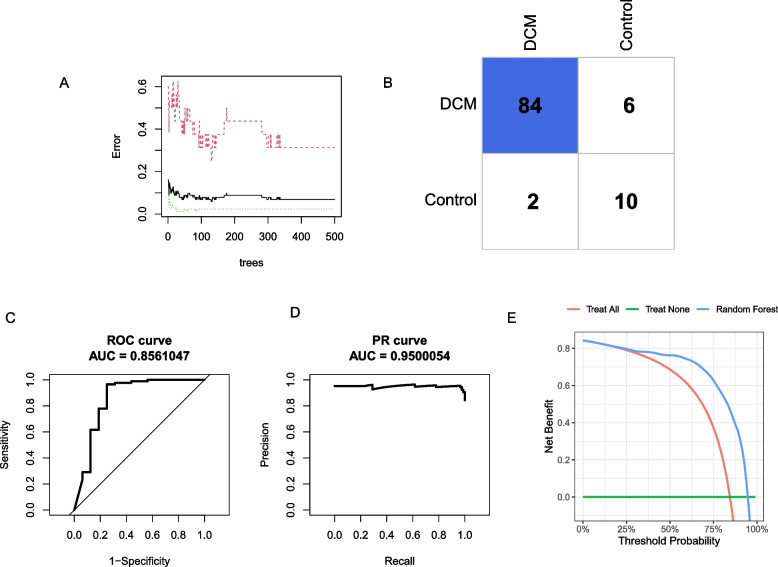
Construction of random forest model. **A** Decision tree quantity screening of random forest model; **B** Confusion matrix of DCM. **C** ROC curve. **D** Precision-recall curve. **E** Decision curve analysis curve

**Fig. 6:**
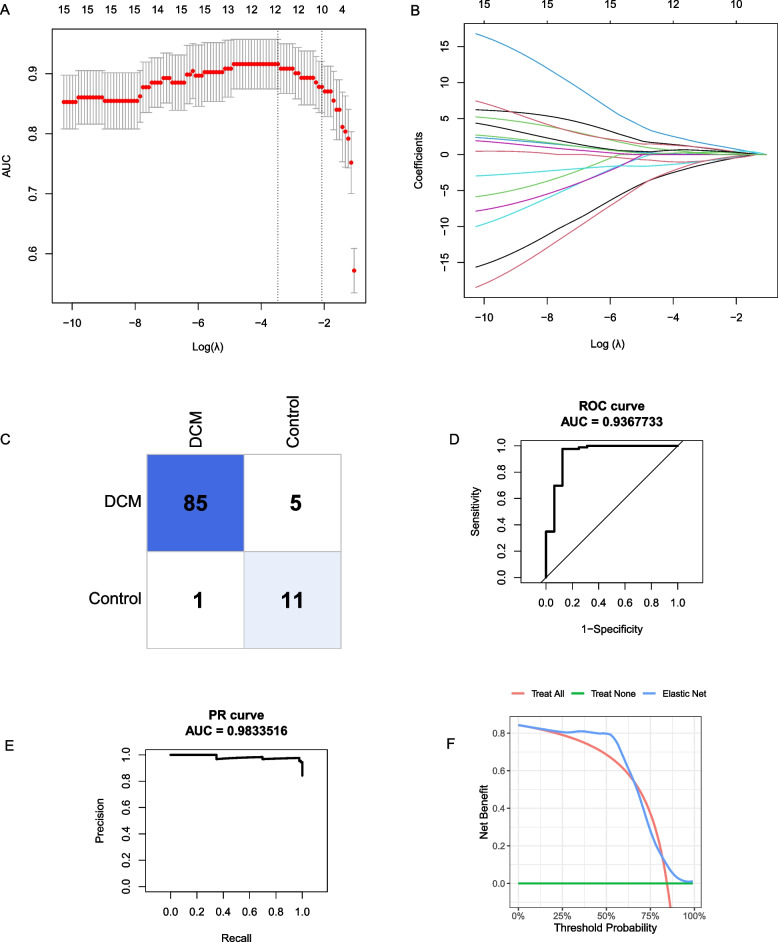
Elastic network model construction. **A**, **B** The process of screening for best values of λ. **C** Confusion matrix of DCM based on elastic network. **D** ROC curve based on EN model. **E** Precision-Recall curve based on EN model. **F** Decision curve analysis curve based on EN model

**Fig. 7:**
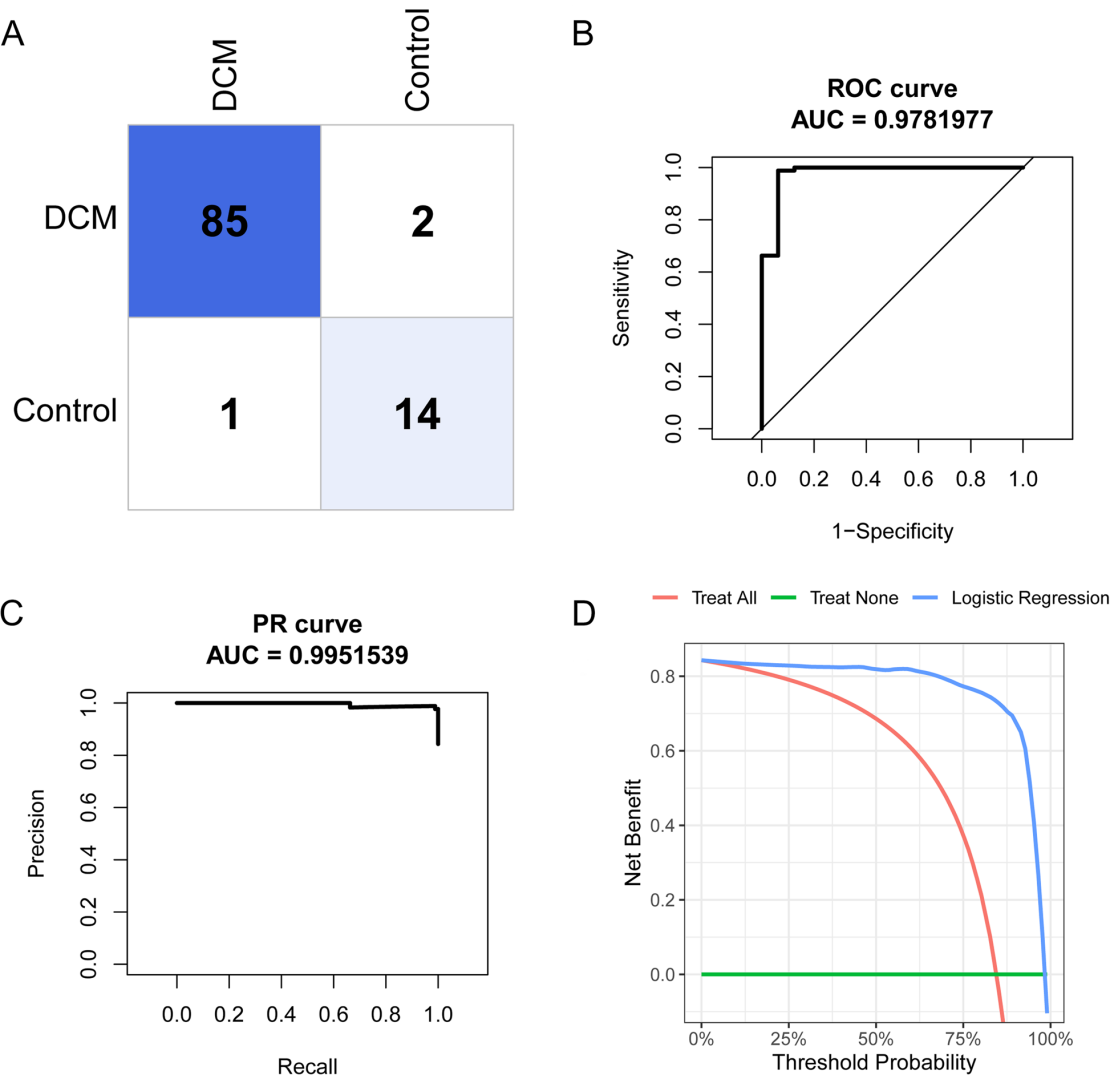
Construction of logistic regression model. **A** Confusion matrix of DCM based on LR model. **B** ROC curve based on LR model. **C** Precision-Recall curve based on LR model. **D** Decision curve analysis curve based on LR model

Then we established three different supervised machine learning models, namely random forest models (RF), elastic network (EN), and logistic regression (LR) models to predict the incidence of DCM. The diagnosis of DCM or not was regarded as the response variable, while the IRDGs were used as explanatory variables. First, the hub genes were adopted to construct a random forest model and the number of decision trees was screened as depicted in Fig. 5A. The Fig. 5 illustrated the lowest classification error when the RF contains 130 decision trees. The model under the optimal number of decision trees was further evaluated in Fig. 5B-E. The RF algorithm achieved a proper diagnosis with an AUC (area under the curve) of 0.86 in ROC and AUC of 0.95 in the PR (precision-recall) curve. In addition, decision curve analysis (DCA) indicated patients could benefit from the RF model at a threshold from 0 to 1. As depicted in Fig. 6A-B, the ideal value of λ belong to 0.031 and the EN model achieved a proper diagnostic value with the AUC (0.94) in ROC and AUC (0.98) in PR curve (Fig. 6C-F). However, the LR model achieved more accurate diagnosis efficiency with the AUC (0.98) in ROC and AUC (0.99) in the PR curve (Fig. 7A-D).

### Comparison and external validation of multiple models

**Fig. 8:**
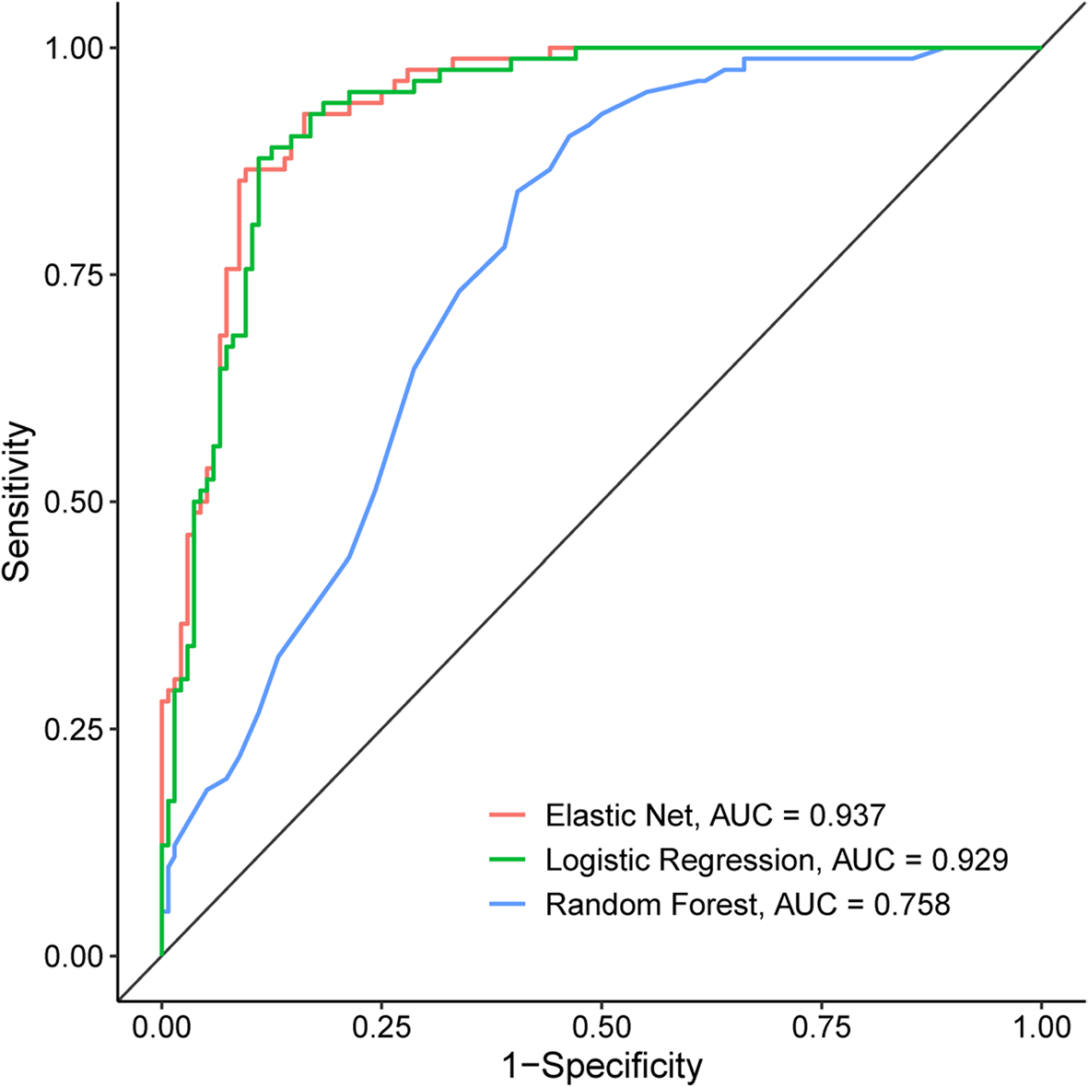
The visual representation of the ROC analysis in GSE57338 based on the three diagnostic models (EN, LR, and RF)

Then we compared the diagnostic effectiveness of different models. Although both EN and LR had higher precision of 0.99, LR had a higher recall than EN and RF. Moreover, LR achieved the highest F1-Score 0.98, and accuracy of 0.97 accompanied by the lowest error of 0.03, which indicated that the LR model earned extraordinary predictive power (Table 2). In addition, the ROC curves of the validation set from GSE57338 also described that the AUC of the EN and LR was 0.937 and 0.929, which was higher than the AUC of 0.758 by the RF algorithms (Fig. 8). Hence, EN and LR analysis based on these hub genes can achieve a better diagnosis of the DCM.

**Table 2:** Comparison of the three diagnostic models

Model	Precision	Recall	F1-score	Accuracy	Error	KS
Logistic Regression	0.99	0.98	0.98	0.97	0.03	0.93
Elastic Net	0.99	0.94	0.97	0.94	0.06	0.85
Random Forest	0.98	0.93	0.95	0.92	0.08	0.72

### Identification of candidate drugs

Moreover, we also explored the potential new drugs based on these IRDGs. The potential drugs were depicted in Table 3, namely suloctidil, acetohexamide, chlorophyllin, prochlorperazine, and arsenenous acid. These drugs had offered new opportunity for the immune intervention in the treatment of DCM.

**Table 3:** The candidate drugs targeting these hub genes

Term	*P*-value	Adjusted *P*-value	Genes
suloctidil HL60 UP	6.39E-19	2.78E-16	ISG20; IFITM1; IFITM2; RSAD2; STAT1 MX1; ADAR; IFIT1; SAMHD1; IFIT3
acetohexamide PC3 UP	5.65E-15	1.23E-12	IFITM1; IFITM2; RSAD2; STAT1; MX1; IFIT1
chlorophyllin CTD 00000324	1.59E-11	2.30E-09	IFITM1; STAT1; MX1; IFIT1; IFIT3
prochlorperazine MCF7 UP	5.05E-10	5.49E-08	ISG20; IFITM1; IFITM2; STAT1; IFIT1
Arsenenous acid CTD 00000922	2.55E-09	2.21E-07	IFITM3;ISG20;IFITM1;IFITM2;RSAD2;STAT1;MX1;HLA-B;HLA-C;IFIT1

## Discussion

DCM refers to the cardiac structural and functional abnormality characterized by dilatation and systolic dysfunction of the left or even the right ventricle, which cannot commonly be attributed to the coronary artery, valvular, and arterial hypertension disorder [29]. It is the third overall cause of HF in the general population and the first cause of heart transplantation worldwide [30]. DCM belong to a comprehensive term which includes numerous inherited or acquired myocardial diseases that result in the same phenotypic outcome such as myocarditis, inflammatory and drug toxicity, autoimmune disease, and gene mutations. The incidence of DCM is often underestimated as the clinical status varies from asymptomatic to end-stage HF [31]. In most cases, the role of inflammation is a natural response to cardiac injury, and a crucial mechanism for healing and tissue repair. However, the abnormal inflammatory response in the heart can always be either inadequate or overwhelming, leading to direct injury or severe host disease. Accumulating evidence has depicted significant inflammatory component in the pathogenesis of DCM, and there were also growing studies reported the relationship between myocarditis and DCM [32, 33]. Our study demonstrated an obvious ratio of immune infiltrating cells between DCM and control donors.

Immunological factors in the pathogenesis of DCM have been proposed based on the demonstration of mononuclear cell infiltration and autoantibodies against the myocardium [34]. According to an authoritative study, all types of immune infiltrating cells ranging from T cells, B cells, macrophages, and granulocytes are increased in hearts with DCM compared with normal hearts, but only CD8 + T cells and macrophages are more prominent than the other leukocyte subpopulations. Moreover, in contrast to normal cardiac tissues, activated makers were detected in approximately 40% of both CD4 + and CD8 + heart-tissue T cells [35]. Our study demonstrated an obvious ratio of T cell CD4 memory activated, T cell regulatory Tregs, and neutrophils between DCM and control donors which was consistent with the previous study [36].

At the same time, our study also identified 274 IRDGs between DCM and control donors. The results illustrated that some genes participated in the positive regulation of cytokine production, response to lipopolysaccharide, and response to molecule of bacterial origin. Abnormal cytokine profiles in patients with idiopathic DCM have previously been reported [37]. Various cytokines were secreted in certain areas from activated inflammatory cells. This may result in a cytokine-rich micro-environment which could exacerbate the enhanced expression of adhesion molecules and thereby contribute to the excessive inflammatory traffic into inflamed myocardial areas [38].

Many factors of DCM coexisted, while different phases of the disease happened simultaneously. There existed three phases during the development of DCM under normal circumstances. Phase 1 is usually characterized by viral infection, autoantigen exposure, or gene mutation, phase 2 mainly manifested with the onset of (probably) multiple autoimmune reactions, and phase 3 by the procession to cardiac dilatation without an infectious agent and cardiac inflammation. The border between the phases is not always distinct, they may overlap one another, and phases 1 and 2 may also recur at the late stage of DCM. The appropriate treatment during phase 1 includes getting rid of the virus and immunomodulatory intervention. Immunosuppression is the most suitable strategy during phase 2 owing to the intense autoimmune processes which calls for sophisticated diagnostic strategies including molecular biological and immunohistochemical techniques. Limited efforts in the phase 3 can be adopted to delay the progression of the disease [39].

At the same time, our study also identified fifteen hub IRDGs (IFITM3, IFITM2, IFITM1, IFIT3, IFIT1, HLA-A, HLA-B, HLA-C, ADAR, STAT1, SAMHD1, RSAD2, MX1, ISG20, IRF2).

Previous studies indicate that DCM can result from an autoimmune disorder according to the Rose-Witebsky criteria. Interferons (IFNs) belong to pivotal cytokines that were involved in immune responses during the activation of hundreds of genes, such as interferon-induced transmembrane proteins (IFITMs). Depart from the convention role of antiviral effects, IFITM take part in a wide range of tumor immunology [40]. Moreover, there were also study demonstrated that IFITM1 and IFITM3 were up regulated during the heart development both in vitro and in vivo study [41]. Our study demonstrated that IFIT family were closely associated with the development of DCM.

The evidence includes the presentation of immune cell infiltration in almost half of the biopsy cardiac samples, abnormal activation of HLA class II and adhesion molecules which are essential to trigger an immune response mediated by HLA class II. The abnormal expression of HLA-II can be used as biomarkers to verify autoimmune response on the cardiac microvascular endothelium regardless of viral genomes from biopsy samples in patients and family members [42]. Moreover, abnormal elevated expression of cardiac autoantibodies in patients serum and experimentally induced DCM animal models with recognized autoantigens can also be tested in patients with DCM before and after treatment of immunosuppression or immunomodulation [43]. Cardiac-specific autoantibodies can be tested in almost 60% of patients with DCM and eventually result in cardiac dysfunction [44]. In patients particularly with gene mutation, inflammatory DCM may be familial and has been associated with HLA antigens, as illustrated by a landmark multi-center European genome-wide association study [45]. The study reports that HLA refers to a risk locus for DCM, similar to autoimmune aetiology and consistent with our research results as the expression of HLA-A, HLA-B, and HLA-C. Previous study demonstrates that various allele of HLA was closely associated with the incidence of both hypertrophic cardiomyopathy and HF [46, 47]. Interferons represent multifunctional cytokines that exert pivotal roles in body defense against viral and parasite infection with the antiproliferative and immune‐modulating effect. IFITM refer to a member of small homologous proteins, localized in the plasma and endolysosomal membranes, which exhibit cellular resistance to many viruses. Apart from the known antiviral effects, elevated expression of IFITM1 and IFITM3 were detected during the heart development [48]. Our research also proved the potential role of the IFITM family.

Obvious downregulation of ADAR2 and increase in ADAR1 expression was observed in blood samples from congenital heart disease (CHD) patients. The downregulation of ADAR2 was in line with its downregulation in ventricular tissues of DCM which was consistent with our study [49]. The signal transduction pathways mediating the progression to HF have been widely investigated both in vitro and in vivo studies. The study discovered that STAT1 were activated in DCM [50], moreover, activation of STAT1 transcription factor precedes up-regulation of coxsackievirus-adenovirus receptor (CAR) during viral myocarditis [51]. SAMHD1was reported as a dNTPase that protects cells against viral infections especially the DNA viruses such as the herpesviruses cytomegalovirus (CMV) and Epstein-Barr virus (EBV), or the hepadnavirus. The Interferon-induced protein with tetratricopeptide repeats (IFIT) family occupied a vital component during the antiviral immune response [52]. IFIT1 belong to one of the interferon stimulated genes (ISGs) family was obviously activated by type I interferon (IFN) and virus infection. IFITKO mice manifested repaid Coxsackievirus group B type 3(CVB3) replication in the heart following infection, while cardiomyocytes constitutive IFIT expression suppresses CVB replication [52]. Moreover, there were also study reported that platelet derived levels of CRP and IL-6 were associated with IFIT1 which may involve in cardiovascular disease risk factors. RSAD2 has been reported as the biomarker both in myocardial injury in dermatomyositis and HF [53], Our study illustrated that RSAD2 was closely associated with the inflammatory response in DCM. Mx1 (myxovirus resistance 1) belong to an important antiviral protein in the innate immune responses of vertebrates to microbial pathogens. Previous study has reported the fatal myocarditis in a patient with anti-MDA5 antibody–positive dermatomyositis accompanied with Myxovirus resistance protein 1 [54].

Early exploration of appropriate therapy is vital in DCM due to the overlap of pathophysiological stages. It requires the precise immunohistochemical and immune gene map of endomyocardial biopsies in parallel. Modern molecular biology advance calls for clear clarification of the infective agent-immune system-host interaction, leading to a deeper understanding of DCM's etiology. During the past two decades, artificial intelligence (AI) has drawn brilliant accomplishment to disease diagnosis and treatment. A great deal of research has been dedicated to predicting the diagnosis and prognosis by using a variety of artificial intelligent approaches. At the same time, the vigorous development of high-throughput biological analysis technology has promoted the remarkable growth of genomic biological data, and the application of informatics research analysis [55]. The systematic analysis in biology transcend the traditional analysis of a single gene or multiple genes at a time, giving rise to a new era of gene research, with which we can identify the gene expression profiles of complex diseases [56]. Gene expression profile provides a key prospect in the prognosis of the disease development. In this study, we have developed three different machine learning methods based on the IRDGs, namely elastic network and logistic regression, and random forest which achieved good diagnostic effects.

Machine learning algorithms give rise to efficient predictive models for the evaluation of cardiovascular disorders such as HF, myocardial infarction, and coronary heart disease. The development of genetic diagnostics can help us better classify and discern patients, thereby applying more accurate treatment strategies and reducing the incidence of end-stage HF. Our study illustrated that all the three models has achieved a proper diagnosis of DCM. With the AUC 0.86 in RF, 0.94 in EN and 0.98 in LR. Despite that both EN and LR had a higher precision of 0.99, the LR had a higher recall than that of both EN and RF. EN and LR models had achieved a proper diagnostic value than that of RF based on these hub genes. Considering the potential role for serious complications and medical resource utilization, DCM prediction is crucial in clinical practice. Machine learning has precipitated multiple studies being conducted to construct predictive models far beyond contemporary guidelines, in which various baseline variables were used with traditional statistical analyses. The clinical implication of the machine learning predictive models based on our study is straightforward. Generally, the model with the highest AUC is recommended, though those with the highest sensitivity or specificity could be appropriate depending on specific conditions.

Researchers have identified a variety of causes of DCM, ranging from genetic mutations, infections, inflammation, autoimmune diseases, toxins exposure, and abnormal endocrine or neuromuscular activation. However, relatively little effort has been made to investigate the potential effects of drugs on DCM. Owing to the disorder of the immune system in DCM, immunotherapy targeting precision medicine came into being, including immunosuppressants and immunoadsorption [57]. An increase in antibodies and a decrease in contractility were observed in DCM 12 months after immunoadsorption therapy and subsequent IgG replacement treatment [58]. Experimental and clinical studies inferred that activation of the humoral immune system, with the production of circulating cardiac autoantibodies, plays an important functional role in the development of DCM. Small open-controlled studies demonstrated that the removal of circulating antibodies by immunoadsorption results in the improvement of cardiac function and relax in myocardial inflammation. At present, immunoadsorption is an experimental treatment option deserved future confirmation by a placebo-controlled multicenter study [59]. Several potential drugs were identified in our study, namely suloctidil, acetohexamide, chlorophyllin, prochlorperazine, and arsenenous acid. Chlorophyllin has been reported as an antioxidant to protect chemically induced breast cancer [60]. There was also emerging immunomodulatory therapeutic strategy has been gradually debated over the past years such as TNF-α Inhibitors and IL-1 Inhibitors. The effectiveness of immunotherapy needs to be further evaluated as well. Moreover, it is worth exploring which kind of patients can gain the maximum benefit from immunotherapy. There were also some limitations about our study. First of all, only two GEO datasets were included in the study. Some bias may exist due to the limited number of sample size. Furthermore, our study identified the DEGs that are primarily immune genes due to the intersection with immune databases which may result in the potential immune bias. As the immune events were closely associated with the entire process of DCM, the overall transcriptome immune relationship may reveal the whole prospect of the incidence of DCM. The immune bias from the DEGs may lead to some immune genes being ignored. Thanks to the rapid development of single cell sequence, there were also some single-cell datasets about the incidence of DCM particular focus on the immune cells [61–63]. The cumbersome machine learning about deconvolution from bulk RNA-sequence may not be required and more precise study from the sing-cell sequence focused on immune analysis were more appropriate. Overall, although some immune bias were existed, the immune genes from the DEGs can also reflect the underline immune events from an immune perspective at some extents.

## Conclusion

In this study, we successfully analyzed IRDGs in patients with DCM and elaborate their potential functions through GO and KEGG pathway analysis. Moreover, under the performance of different supervised machine learning models, we established logistic regression and elastic network diagnostic models of DCM based on these hub IRDGs in DCM and verified the effectiveness with another data set. At the same time, our study also preliminary identified the potential drugs targeting these genes which could be useful for the early diagnosis and drug development.

## Data Availability

All the datasets analysed during the current study are available in the NCBI-GEO database (https://www.ncbi.nlm.nih.gov/geo/ GSE5406 and GSE57338). The data that support the findings of this study are available from the corresponding author, Haihua Chen, upon reasonable request.

## Abbreviations

ADAR: Adenosine deaminase acting on RNA
AI: Artificial intelligence
BNP: Brain natriuretic peptide
BP: Biological process
CC: Cell component
CD: Cluster of differentiation
CVB3: Coxsackievirus group B type 3
Cys-C: Cystatin C
CHD: Congenital heart disease
cTnI/TnT: Cardiac troponin I/T
DAMPs: Damage-associated molecular patterns
DCA: Decision curve analysis
DCM: Dilated cardiomyopathy
DEGs: Differentially expressed genes
EBV: Epstein-Barr virus
EMB: Endomyocardial biopsy
EN: Elastic network
FITMs: Interferon-induced transmembrane proteins
GDF-15: Growth differentiation factor-15
GEO: Gene expression omnibus
CMV: Cytomegalovirus
GO: Gene ontology
HF: Heart failure
H-FABP: Heart type-fatty acid binding proteins
HLA-A: Human leukocyte antigen- A
HLA-B: Human leukocyte antigen- B
HLA-C: Human leukocyte antigen- C
IL-1: Interleukin-1
IL-2: Interleukin-2
IL-6: Interleukin-6
IFITM: Interferon-inducible transmembrane
IFITM1: Interferon-induced transmembrane protein 1
IFITM2: Interferon-induced transmembrane protein 2
IFITM3: Interferon-induced transmembrane protein 3
IFIT1: Interferon-induced protein with tetratricopeptide repeats 1
IFNs: Interferons
IRF2: Interferon regulatory factor 2
IRDGs: Immune-related differential genes
IRGs: Immune-related genes
ISGs: Interferon stimulated genes
ISG20: IFN-stimulated exonuclease gene 20
KEGG: Kyoto encyclopedia of genes and genomes
LR: Logical regression
LVEF: Left ventricular ejection fraction
MF: Molecular function
MHC: Major histocompatibility complex
MHC II: Major histocompatibility class II
MR-proADM: middle regional adrenomedullin
MX1: Myxovirus resistance 1
NK: Natural killer
NLR: Neutrophil to lymphocyte ratio
NYHA: New York Heart Association
PAMPs: Pathogen-associated molecular patterns
PCA: Principal component analysis
PPI: Protein–protein interaction
PR: Precision-recall
RF: Random forest model
ROC: Receiver operating curve
RSAD2: Radical S-adenosyl methionine domain-containing protein 2
SCD: Sudden cardiac death
sST2: Soluble ST2
STAT1: Signal transducer and activator of transcription 1
SAMHD1: Sterile α motif and HD domain-containing protein 1
Th: T-helper (Th)
TLRs: Toll-like receptors
TNF-α: Tumor necrosis factor-α
VA: Ventricular arrhythmia
